# USP8 promotes the tumorigenesis of intrahepatic cholangiocarcinoma via stabilizing OGT

**DOI:** 10.1186/s12935-024-03370-w

**Published:** 2024-07-07

**Authors:** Guo Long, Dong Wang, Jianing Tang, Kuan Hu, Ledu Zhou

**Affiliations:** 1 Department of Liver Surgery, Xiangya Hospital, Central South University, Changsha, 410008 Hunan China; 2 National Clinical Research Center for Geriatric Disorders, Xiangya Hospital, Central South University, Changsha, 410008 Hunan China; 3 Liver Disease Center, The Affiliated Hospital of Qingdao University, Qingdao, 266000 Shandong China

**Keywords:** USP8, Intrahepatic cholangiocarcinoma, OGT, Ubiquitination, Pemigatinib

## Abstract

Ubiquitination was considered to be a crucial factor in intrahepatic cholangiocarcinoma (iCCA) development. Herein, we identified Ubiquitin-specific peptidase 8 (USP8) as a key regulator for promoting the tumorigenesis of iCCA cell via stabilizing OGT. USP8 was overexpressed in human tumor tissues and correlated with worse survival. Moreover, the mass spectrometry and co-immunoprecipitation analysis indicated that USP8 interacted with OGT. USP8 worked as a bona fide deubiquitylase of OGT. It stabilized OGT in a deubiquitylation activity-dependent manner. Meanwhile, DUB-IN3, the USP8 inhibitor, could also restrain the malignancy of intrahepatic cholangiocarcinoma. In addition, USP8 depletion promoted the response of iCCA to pemigatinib. In conclusion, our findings pointed to a previously undocumented catalytic role for USP8 as a deubiquitinating enzyme of OGT. The USP8-OGT axis could be a potential target for iCCA therapy.

**Supplementary Information:**

The online version contains supplementary material available at 10.1186/s12935-024-03370-w.

## Introduction

Intrahepatic cholangiocarcinoma (iCCA) is the second most common primary liver cancer (PLC), only after hepatocellular carcinoma [1, 2]. The incidence of iCCA rises significantly throughout all ages and races in eastern and western countries [2–4]. iCCA is defined as CCA above the hilar junction of bile ducts, whereas extrahepatic cholangiocarcinoma (ECC) develops within or below the hilum [5–7]. The prognosis for iCCA remains grim despite recent improvements in therapy methods. iCCA frequently owned fibroblast growth factor receptor (FGFR) 2 fusion gene aberrations or isocitrate dehydrogenase 1 or 2 (IDH1 or 2) gain of function genetic mutations. Genetic alterations of FGFR2 or IDH1/2 have implications for therapy. Therefore, the targeted therapy was vital in iCCA. Recently, a new small molecule inhibitor of FGFR2, pemigatinib, received accelerated approval by US FDA, bringing a potential treatment for iCCA patients [8–10]. Recent clinical trials have indicated that pemigatinib can significantly prolong survival in advanced iCCA [8, 9]. However, the response of iCCA to pemigatinib remains suboptimal [11]. Therefore, it is urgent to improve the efficiency of iCCA to pemigatinib.

Ubiquitination is an essential posttranslational protein modification that regulates many eukaryotic signaling pathways [12]. It is known that abnormal ubiquitin signaling is a biological cause of certain cancers, neurological illnesses, immune system disorders, and cardiovascular ailments [13–16]. Ubiquitination can be reversed by distinct kinds of deubiquitinase [17]. Deubiquitinating enzymes (DUBs) prevent proteins from degrading by removing ubiquitin from protein substrates. As a result, free ubiquitin is liberated and takes part in the cycle of ubiquitination [18, 19]. Ubiquitin-specific peptidase 8 (USP8), a member of DUB, regulates various substrates such as SQSTM1, P27, ESCRT-III, and c-Met in cancers [20–24]. However, the pathophysiology and functions of USP8 have not been thoroughly verified in iCCA [25].

O-GlcNAc transferase (OGT) is a vital regulator in O-linked β-N-acetylglucosamine modification (O-GlcNAcylation) [26]. Accumulating evidence has revealed that abnormal expression of OGT forms a cross-link between epigenetics and tumorigenesis [27]. However, the upstream role of OGT in cancer biology is poorly characterized. In this study, we unexpectedly find that USP8 could deubiquitylate and stabilize OGT in a deubiquitylation activity-dependent manner. We further demonstrate USP8 promotes tumorigenesis and impacts on the sensitivity of iCCA to pemigatinib. Our findings provide potential therapeutic opportunities for iCCA by targeting USP8-OGT axis.

## Materials and methods

### Patients specimens and IHC analysis

Paraffin-embedded tissues including tumors and paired normal tissue were collected from 126 iCCA patients who underwent hepatectomy at Xiangya hospital between 2015 and 2020. IHC staining were performed on 5 μm–thick paraffin sections. The quantification of IHC staining was done according to the proportion of positively stained tumor cells and the intensity of staining. The proportion of positively stained tumor cells was graded as follows: 0, no positive tumor cells; 1, 0.01%–25% positive tumor cells; 2, 25.01%–50% positive tumor cells; 3, 50.01%–75% positive tumor cells; and 4, 75% or greater positive tumor cells. The cells at each intensity of staining were recorded on a scale of 0 (no signal), 1 (weak), 2 (moderate), and 3 (strong). The IHC score for each section was computed by the following formula: IHC score = staining intensity × proportion of positively stained tumor cells. In compliance with the Declaration of Helsinki, all patient data were collected anonymously. This study was authorized by the Ethics Council of Xiangya Hospital affiliated to Central South University (No. 202103377).

### IP mass spectrometry and RNA sequencing assays

For IP mass spectrometry assay, HCCC9810 cells (three samples were prepared) was wash by PBS for three times. Then, we used the RIPA lysis buffer (Abiowell, China) which contained a cocktail of protease inhibitors to lyse the cells. Rabbit IgG was used to preclear the total cell lysis buffer for 4 h, followed by an overnight immunoprecipitation along with USP8 antibody and Protein A/G PLUS-Agarose beads at 4 °C. The immunoprecipitated proteins were collected for LC-MS mass spectrometry. And technical support was provided by oebiotech company (www.oebiotech.com). For RNA sequencing assay, we first transfected FGFR- CCDC6 fusion plasmid into HCCC9810 cells. Then, HCCC9810 cells were treated by pemigatinib or DMSO for one month, respectively. Then, we obtained HCCC9810 resistant (HCCC9810-R) cell line and HCCC9810 sensitive (HCCC9810-S) cell line. Next, we extract the total RNA from the cells by using Hipure total RNA mini kit (Magen, Guangzhou, China) according to the manufacturer’s instructions. Finally, the total 6 samples (3 samples for HCCC9810 resistant cell and 3 samples for HCCC9810 sensitive cell) were used for RNA sequencing and technical support was provided by HUADA company (www.genomics.cn). The results of RNA sequencing were in Table S3.

### Cell culture, plasmids, shRNAs, drugs and chemicals

The human intrahepatic cholangiocarcinoma cell lines HCCC9810 and RBE were purchased from American Type Culture Collection (ATCC). RPMI-1640 (Cellmax, China) with 10% fetal bovine serum was used to culture HCCC9810 and RBE under 37 °C and 5% CO2 culture conditions. USP8 cDNA clones were purchased from Vigene Biosciences. Then, the Flag-USP8 plasmid was established by insertion of the USP8 cDNA into the Plvx-flag vector. USP8 overexpression, Wild-type (WT) USP8 and the inactive mutant plasmids were purchased from Hanbio Biotechnology (Shanghai, China). Plasmid transfection using Lipofectamine^®^ 2000 was carried out according to the manufacturer’s instructions. The sequences of shRNAs targeting USP8 were 5′- GCTGTGTTACTAGCACTATAT -3′ (#1) and 5′-GCTGTGTTACTAGCACTATAT -3′ (#2). DUB-in3 (HY-50737-11866) and MG132 (HY-13259-110290) were purchased from MedChemExpress in vivo experiments. Besides, for explored the response of iCCA cells to Pemitibib, we used the FGFR2-CCDC6 fusion plasmids to transfect iCCA cells. The sequence was 5′-AGGACCGGGGATTGGTACCGTAAC-3′. The details about the reagents were listed in Table S4.

### Cell proliferation and migration assays

The cell Counting Kit-8(CCK8) and EdU incorporation assay were used to detect cell viability. Briefly, 1 × 10^3^ cells were seeded into duplicate wells of 96-well plates for CCK8 assay. After the mixture with CCK8 reagent for 1 h, we measured the OD470 at the same time every 24 h. For EdU incorporation assay, we used a 24-well plate to culture iCCA cells. After cells grew about 70% of the well, we performed the EdU assay by the instructions. The wound healing assay was performed to assess the cell migration capacity. HCCC9810 and RBE cells were planted in 6 well dishes. When cells grew almost covering the dish, a 200 μl pipette tip was used to scratch on the cell. Cells were cultured by 1640 medium with 1% FBS. The wound width was measured at the same time for each day. The transwell assay was performed as follow. Matrigel and serum-free culture media were mixed in a 1:7 ratio. The 50 μl mixture was added to the chamber. Each chamber was seed 5 × 10^5^ cells. Then, the cells were fixed with methanol and stained with 0.5% crystal violet 48 h later. A microscope was used to count the cells.

### Animal study

Female SCID mice aged four weeks were purchased from Hunan SJA Laboratory Animal Co., Ltd. (Changsha, China) for the xenograft tumor model. Animal protocols were approved by the Ethics Committee at Xiangya Hospital of Central South University. HCCC9810 cells were dissociated with trypsin and washed with PBS. Then, stable USP8-overexpressing or USP8-deletion HCCC9810 cells, as well as the equivalent control cells, were injected subcutaneously into the axilla or inguinal area of each mouse (5 × 10^6^ cells/mouse). Besides, Tumor-bearing mice were pooled and randomly divided into the following groups: (1) vector; (2) USP8 inhibitor (DUBs-IN-3); (3) USP8-OE; (4) USP8-sh; (5) USP8-shctr. All treatments were conducted by intraperitoneal injection every three days.

To explore whether USP8 affected tumor growth in vivo, we performed a xenograft model assay. In brief, 5 × 10^6^ cells shControl or shUsp8 HCCC9810 cells in 100 μl PBS were subcutaneously injected into the flank of 4-week-old female SCID mice, respectively. On the day of 3 after tumor cells injection, tumor sizes were measured every day. At the end of the fourth week, the tumours were harvested and weighed.

To explore whether USP8 inhibitor affected tumor growth in vivo, we performed a xenograft model assay. In brief, 5 × 10^6^ cells HCCC9810 cells in 100 μl PBS were subcutaneously injected into the flank of 4-week-old female SCID mice, respectively. On the day of 3 after tumor cells injection, tumor-bearing mice were pooled and randomly divided into the following groups: (1) vector; (2) USP8 inhibitor. Tumor sizes were measured every day. At the end of the fourth week, the tumours were harvested and weighed. The USP8 inhibitor treatment was given with a dosage of 2 mg/kg of mouse body weight by intraperitoneal injection every 3 days.

All the animal study was authorized by the Animal Ethics Council of Xiangya Hospital affiliated to Central South University (No. 202103378).

### Western blot analysis and co-immunoprecipitation assay

PBS was used to wash the iCCA cells before lysate them in IP lysis solution (Abowell. AWB0164, China) with a protease inhibitor cocktail on ice. The protein of the sample was electrophoresed in 10% sodium dodecyl sulfate–polyacrylamide gel. Then, the proteins were transferred to a polyvinylidene fluoride membrane (Millipore, USA). For immunoprecipitation experiments, the sample was prepared as above. Rabbit IgG was used to preclear the total cell lysis buffer for 4 h, followed by an overnight immunoprecipitation along with either USP8 (Cell Signaling Technology, #8644) or OGT (Proteintech, 10851-1-AP) antibody. Finally, western blotting was used to analyze the samples. The western blotting analysis were performed in in three biological replicas/each.

### In vivo deubiquitination assay

HCCC9810 cells and HEK293 cells were used to perform the vivo deubiquitination assay. HA-Ub, Flag-USP8, Myc-OGT, or Myc-USP8 plasmids were transfected into HEK293 cells for two days. HCCC9810 cells with USP8 depletion were transfected with HA-Ub plasmid. Western blotting was used to analyze the ubiquitination of OGT.

### Immunofluorescence assay

HCCC9810 and RBE cells were cultured on 15 mm coverslips on 12 well plates. Then, the cells were fixed using 4% paraformaldehyde. PBS was used to wash the fixed cells three times. To block the cells for 24 h, 10% goat serum with USP8 and OGT antibodies was used. At last, the fixed cells were incubated with FITC and Cy3-conjugated secondary antibodies. The Leica N2 microscope was used to examine images.

### Statistical analysis

Student’s t-test and one-way ANOVA were used to compare two and more groups respectively. When necessary, multiple comparisons with Bonferroni correction were carried out. A *P* value < 0.05 was considered statistically significant. All statistical tests were performed with Prism 7.0 (GraphPad, USA).

## Results

### USP8 impacted the sensitivity of iCCA to pemigatinib

**Fig. 1:**
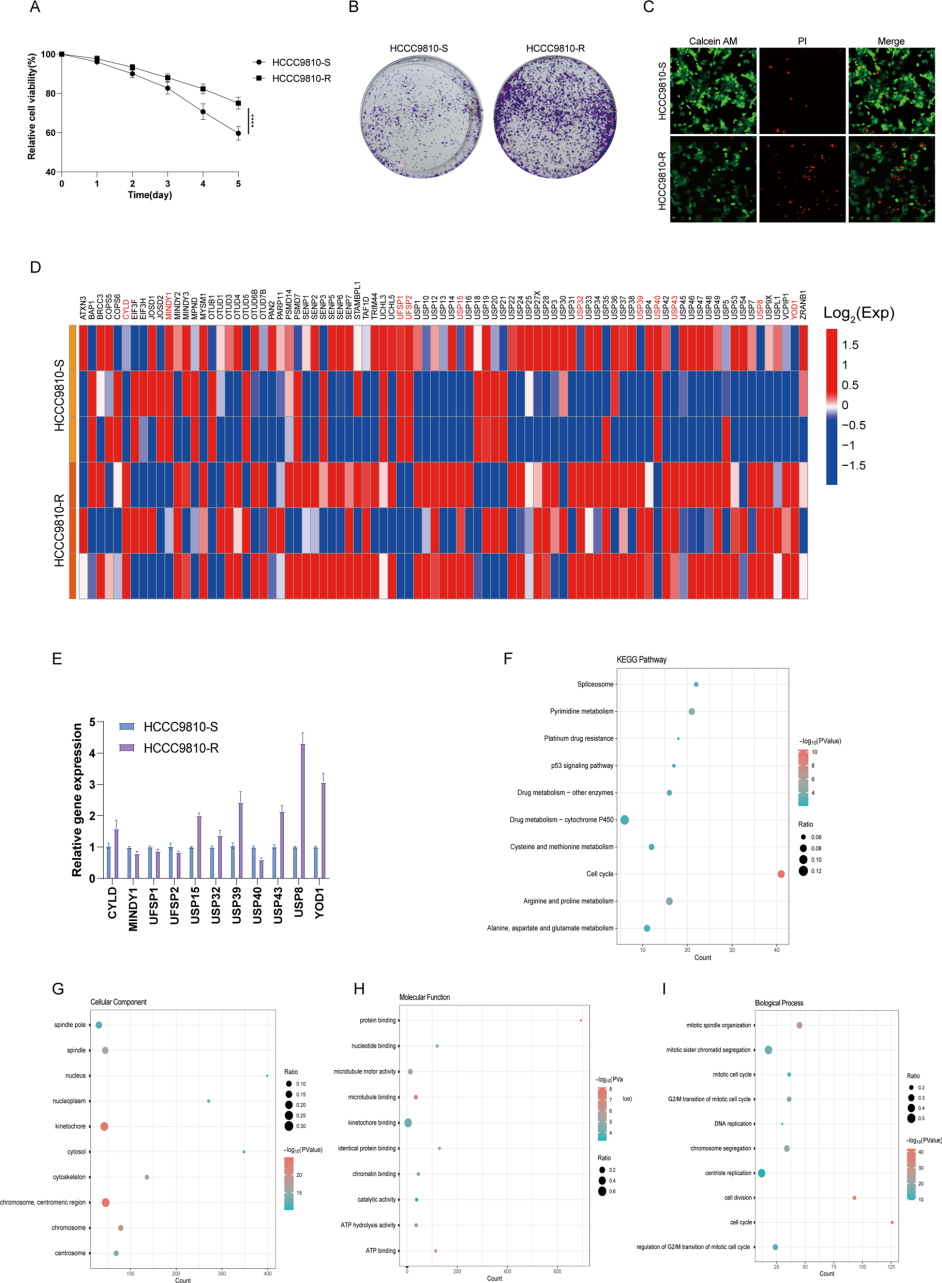
USP8 was elevated in iCCA and linked to a poor prognosis. **A** HCCC9810-R cell showed better proliferation ability by CCK8 assay, HCCC9810 cell was transinfected FGFR2 CCDC6 fusion plasmid. **B** and **C** Crystal violet staining and Edu assay were used to examine the cell viability under the pemigatinib treatment. **D** The total RNA was isolated from HCCC9810-S and HCCC9810-R cells. The heat map showed the differences of RNA sequence between the two cell lines. **E** USP8 gene expression showed the largest difference between the two cell lines. **F** KEGG Pathway analysis between the two kinds of cells. **G**, **H** and **I** GO enrichment analysis showed the BP, CC, and MF of the two kinds of cells

To explore whether the DUBs impacted the sensitivity of iCCA to pemigatinib, HCCC9810 cells which transfected with FGFR CCDC6 fusion plasmid were treated by pemigatinib (10.0 nM) or DMSO for one month, respectively. Then, we obtained HCCC9810 resistant (HCCC9810-R) cell line and HCCC9810 sensitive (HCCC9810-S) cell line. The CCK-8, crystal violet staining and EdU assays indicated the HCCC9810-R cell had a better growth and survival capacity than the HCCC9810-S cell line under the treatment with pemigatinib (Fig. 1A, B, and C). Furthermore, we extracted the total RNA from each cell line and performed RNA sequence analysis to identify the differentially expressed DUBs (Fig. 1D). As the results showed, USP8 was the most significantly upregulated among all detected DUBs (Fig. 1E). KEGG Pathway analysis showed that the differentially expressed genes of HCCC9810-R and HCCC9810-S cell lines were enriched in drug metabolism pathways (Fig. 1F). We used the data of RNA sec to perform the gene ontology analysis. The GO analysis depicted the cellular component, molecular function and biological process. The cellular component (CC) showed that the HCCC9810-R cell was primarily correlated with kinetochore binding (Fig. 1G). Formolecular function (MF) showed that kinetochore binding was mainly enriched for the HCCC9810-R cell (Fig. 1H). The biological process (BP) indicated the enrichment function of the centrione replication process and mitotic sister chromatid segregation (Fig. 1I). These results indicated that USP8 impacted the sensitivity of iCCA to pemigatinib.

### USP8 associated with a poor prognosis in iCCA patients

**Fig. 2:**
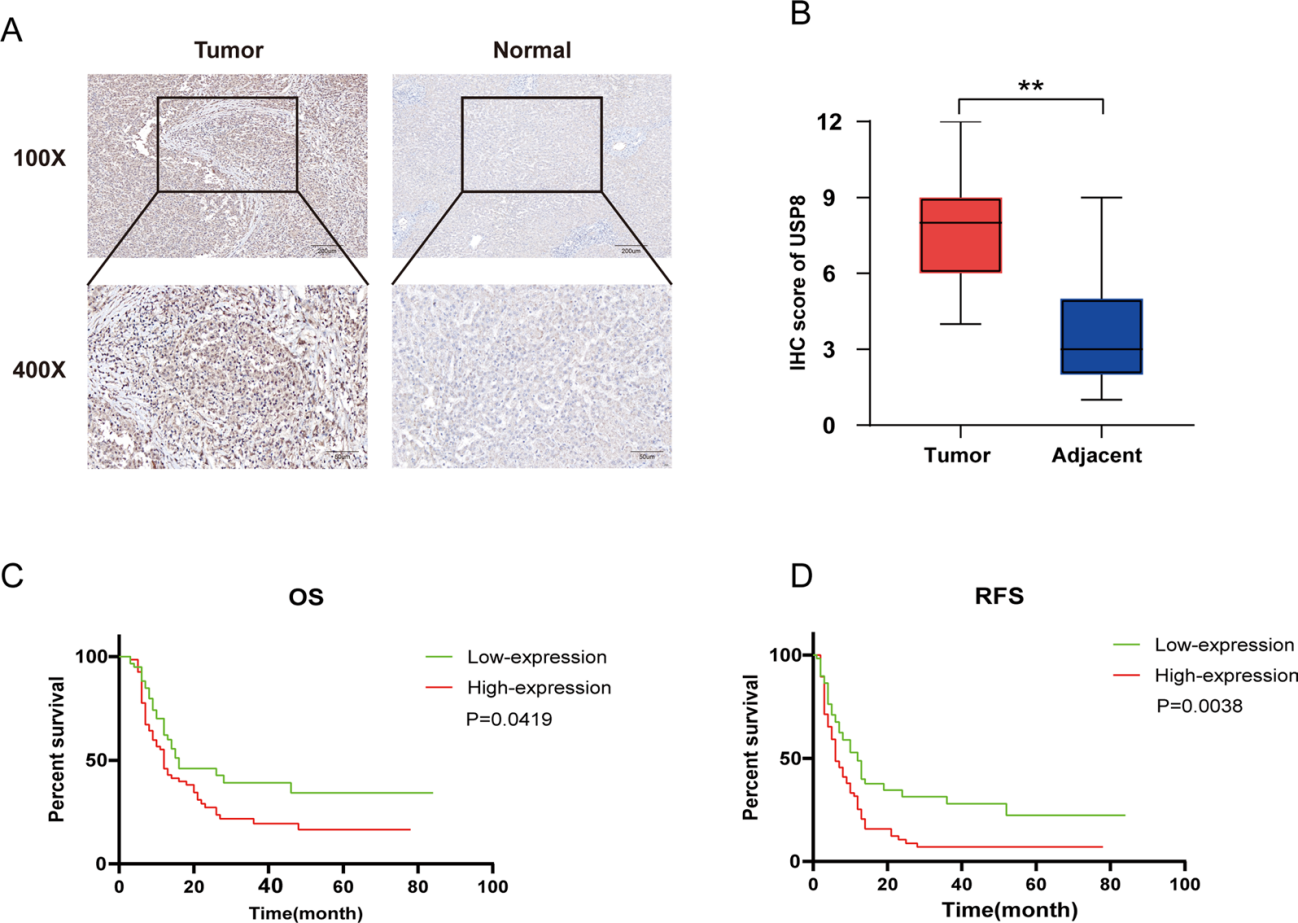
USP8 associated with a poor prognosis in iCCA patients. **A** and **B** USP8 expression levels in tumor tissue and adjacent normal tissue were analyzed using IHC. **C** and **D** The analysis of OS and PFS in USP8 high expression group and low expression group. *P < 0.05, **P < 0.01, ***P < 0.001

To illustrate the role of USP8 in iCCA, we first explored the expression of USP8 in clinical samples. Immunohistochemistry (IHC) staining was performed in tumor tissues and matched normal tissues of 126 iCCA patients. Upregulation of USP8 was observed in tumor tissue (Fig. 2A, B). Moreover, we utilized the Kaplan–Meier analysis to assess the correlation between USP8 expression levels and survival. The results showed that upregulation of USP8 was associated with poor prognosis (Fig. 2C, D).

Then, we investigated the relationship between the expression of USP8 and clinical features in iCCA patients. According to the IHC score, all 126 iCCA patients were separated into two groups. The results revealed that increased USP8 expression was positively associated with tumor size and TNM stage but did not relate to age, gender, CA19-9, hepatitis infection, tumor differentiation, or lymphatic metastasis (Table S1). In summary, these findings indicated that increased USP8 expression was strongly associated with tumor malignancies and a worse prognosis.

### USP8 interacted with OGT and enhances OGT stability

**Fig. 3:**
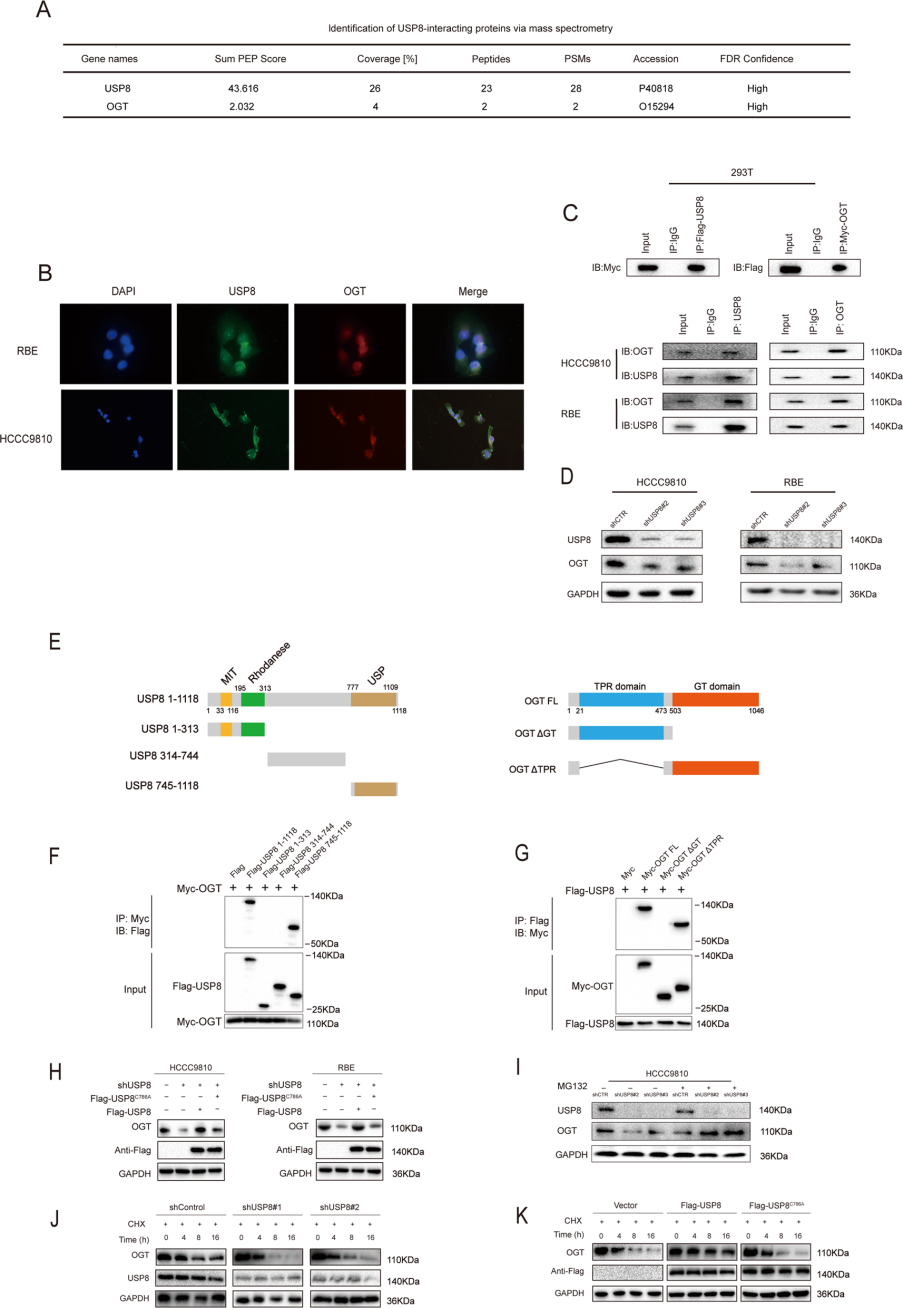
USP8 interacts with OGT and enhances OGT stability. **A** Mass spectrometry analysis revealed USP8 interacted with OGT. **B** The immunofluorescence images showed that USP8 and OGT at colocalized in HCCC9810 and RBE cells. **C** IP identification revealed a bonding between exogenous and endogenous USP8 and OGT in HEK293, HCCC9810 and RBE cells. **D** Western blot analysis revealed depletion of USP8 inhibited the expression of OGT. **E** The structure of the USP8 and OGT domains as well as deletion mutants were constructed. **F** USP8 regulated OGT through the USP domain. USP8 full-length or mutations were transfected into HEK293 cells along with 2 μg Myc-OGT. Using NP-40 lysis buffer, cells were collected after 24 h. Myc antibody was used for co-IP. The possible interacted USP8 domains were detected by Flag antibody. The Flag antibody was used to find the potential interacting domains of USP8. **G** OGT interacted with USP8 via its GT and TPR domains. OGT full-length or mutants were transfected into HEK293 cells along with 2 μg Flag-USP8. Using NP-40 lysis buffer, cells were collected after 24 h. Flag antibody was used for co-IP. The Myc antibody was used to find the potential interacting domains of USP8. **H** USP8 (wild type or C786) was transfected into iCCA cells with USP8 depletion. The OGT level was measured. **I** The expression of OGT was not further decreased by USP8 depletion when the proteasome inhibitor MG132 was present. HCCC9810 cells were transfected with shUSP8 or shControl RNA. Cells were treated with 10 mM MG132/vehicle for 6 h after 2 days, and then lysates of the treated cells were prepared for western blot analysis. **J** knockdown USP8 decreased OGT half-life. iCCA cells (HCCC9810 and RBE) were transfected with siUSP8 or siControl. After 48 h, cells were treated with 100 μM cycloheximide/vehicle for indicated times. Cell lysates were prepared for western blot analysis. **K** USP8^C786A^ lost the ability to increase half-life of OGT in HEK293 cells. OGT, Myc-tag, Flag-USP8, and USP8C786A plasmids were all transfected into HEK293 cells. Cells were exposed to 100 μM cycloheximide/vehicle for the specified periods after 24 h

We found that USP8 was closely correlated with iCCA prognosis, indicating that it might play a crucial role in the development of iCCA. Then, protein mass spectrometry was performed to investigate the deep mechanism of USP8 in the tumor (Supplementary Table S2). We surprisingly found OGT was immunoprecipitated by USP8 (Fig. 3A). We further explored the interrelation between USP8 and OGT. The immunofluorescence experiment revealed USP8 and OGT were both localized in the nucleus of iCCA cells (Fig. 3B). Moreover, the co-immunoprecipitation (IP) assay revealed that USP8 could interact with OGT in 293 T cells. Besides the association between endogenous USP8 and OGT was also identified in HCCC9810 and RBE cells. (Fig. 3C). Besides, western blotting analysis revealed that knockdown of USP8 dramatically reduced OGT protein levels (Fig. 3D). The above findings indicated that USP8 was a regulator of OGT expression in iCCA.

As previous studies indicated USP8 was a member of the family of ubiquitin-specific proteases (USPs) [28]. It harbored three functional domains. The Rhodanese homology (RH) domain and the trafficking (MIT) domain were located on the N-terminal side of USP8. Furthermore, the catalytic domain of USP8 was positioned on the C-terminal side. Therefore, we constructed the deletion mutants of USP8 as follows: MIT domain and RH domain (USP8 1-313), other domain (USP8 314-714), and USP domain (USP8 715-1118). Meanwhile, OGT deletion mutants were created that lacked each of the component domains (TPR domain and GT domain) (Fig. 3E). Then, we performed the Co-IP assay to explore the interaction domains of OGT and USP8. The results indicated that the USP domain was required for interaction with OGT (Fig. 3F). Besides, the GT domain had the ability to interact with USP8 (Fig. 3G). The investigation of USP8 mutations demonstrated that the base at position 786 was an essential for USP8 and its deubiquitinating [22]. We re-expressed wild-type USP8 and the inactive mutants USP8 to reverse the knockdown effects in HCCC9810 and RBE cells with USP8 depletion. USP8 depletion obviously decreased OGT expression, and the reduced expression of OGT could be reversed by the re-expression of wild-type USP8 (Fig. 3H).

We next performed a series of assays to assess OGT protein stability. HCCC9810 cells with USP8 depletion were treated by proteasome inhibitor MG132. The results demonstrated a notable inhibition of OGT degradation when MG132 was present (Fig. 3I). Moreover, OGT protein degradation level was determined under the treatment of CHX. In HCCC9810 cells with USP8 depletion, the half-life of OGT was shortened (Fig. 3J). As opposed to the catalytically inactive mutant USP8^C786A^, the half-life of OGT was significantly lengthened in cells overexpressing the wild-type USP8 (Fig. 3K). These results demonstrated that USP8 increased OGT stability.

### USP8 stabilized the OGT protein through deubiquitination

**Fig. 4:**
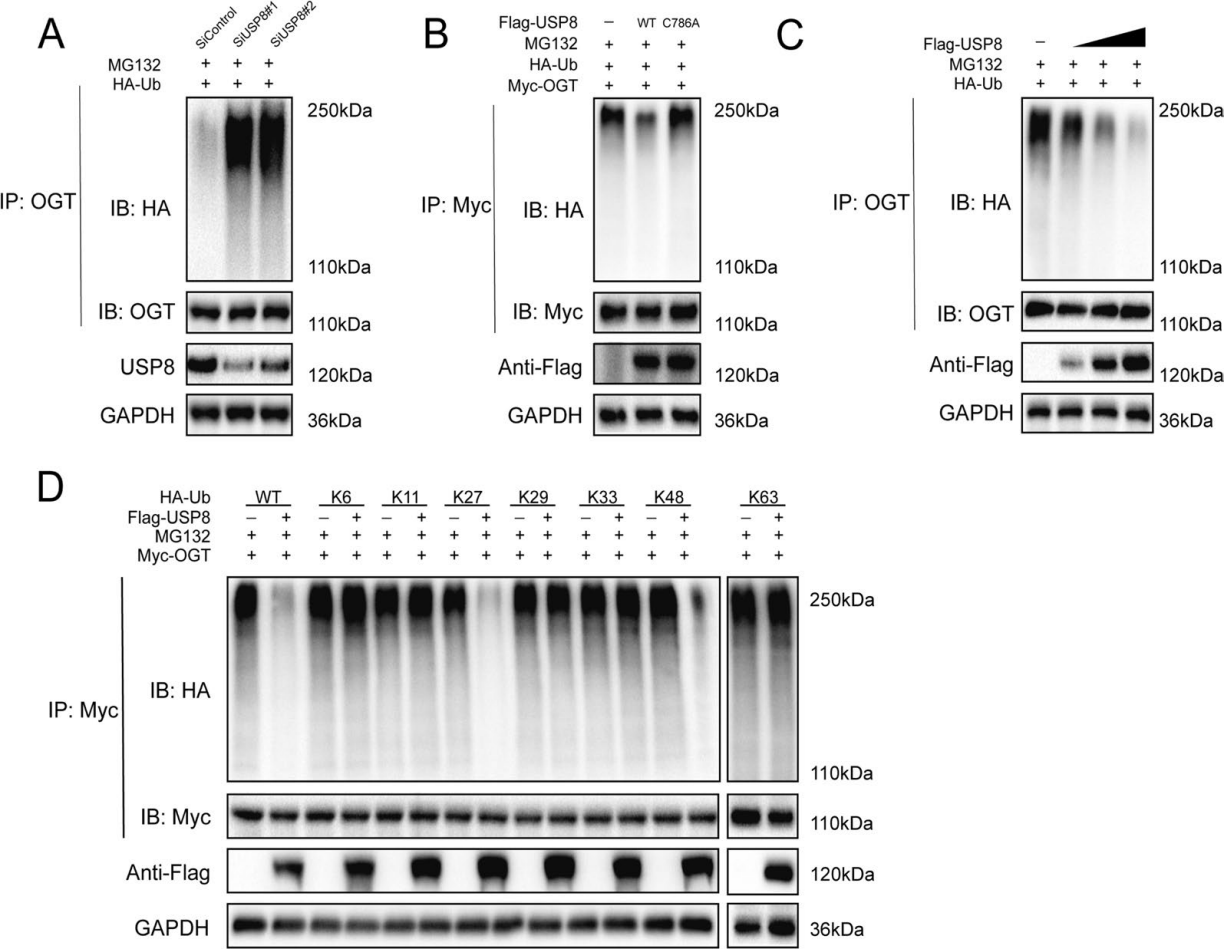
USP8 stabilized the OGT protein through deubiquitination. **A** OGT protein was degraded through deubiquitination when USP8 was inhibited and was exposed to MG132 for 6 h before collection. Anti-OGT was used to immunoprecipitate OGT, while anti-HA was used for immunoblotting. **B** In HEK293 cells cotransfected with Myc-OGT, HA-Ubiquitin, and Flag-USP8 (wild type or C786A), immunoblotting was utilized to identify the ubiquitination of OGT. **C** In a time- and dose-dependent way, USP8 eliminated the OGT ubiquitin chain. **D** USP8 removed the K11- and K48-linked ubiquitin chains on OGT. HCCC9810 cell lysates were used for the ubiquitination experiment after 6 h of treatment with 10 M MG132. Then, the HA antibody was used to determine the ubiquitination level of OGT. Each WB assay was repeated three times to verify our conclusions

In this section, we discovered depletion of USP8 raised the degree of ubiquitinated OGT (Fig. 4A). Meanwhile, the ubiquitylation of OGT was significantly reduced by ectopic production of USP8-WT whereas USP8C786A did not (Fig. 4B). Then, we detected the ubiquitination level of OGT in cells treated with different doses of USP8 plasmids. The results indicated that USP8 directly eliminated the ubiquitin chain of OGT in a dose-dependent way (Fig. 4C). We further carried out ubiquitination assays with a variety of ubiquitin mutants to determine which kind of ubiquitin chain of OGT was affected by USP8. It was found that USP8 could remove the K27 and K48 linked ubiquitin chains from the OGT protein (Fig. 4D). Generally, the above findings indicated that USP8 was a DUB of OGT. Meanwhile, USP8 promoted OGT protein stability in a DUB activity-dependent way.

### Modulation of USP8 affects cell growth, colony formation, and tumor formation

**Fig. 5:**
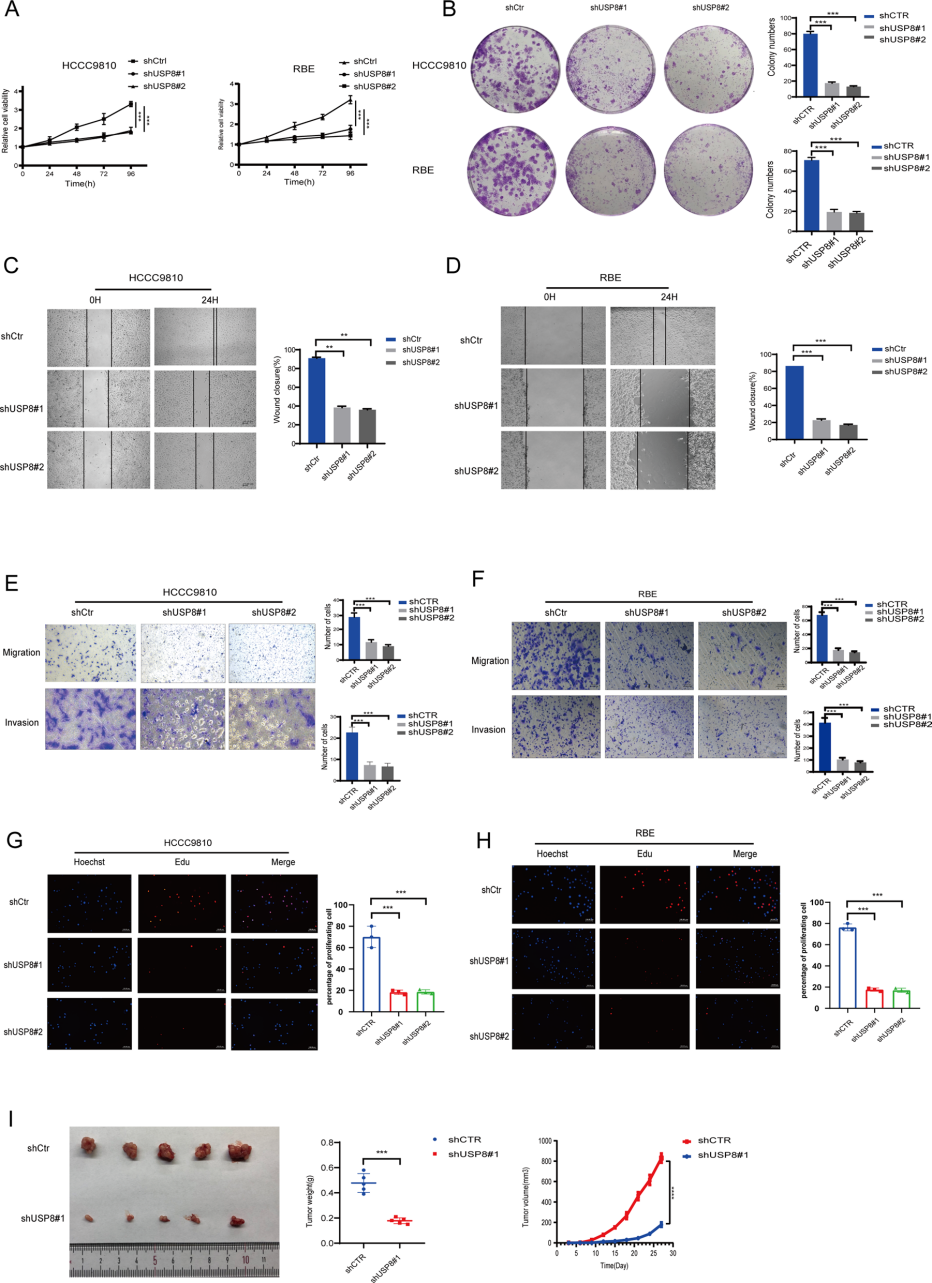
Knockdown of USP8 inhibited cell growth, colony formation, and tumor formation. **A** The vitality of cells was evaluated using the CCK8 assay in iCCA cells with USP8 depletion. **B** USP8 depletion decreases clone formation ability in iCCA cells. **C**, **D** Wound-healing assay in iCCA cells. HCCC9810 and RBE cells with USP8 depletion or not were seeded into 6-well plates. Cells were cultured with 1% FBS. Using a 200 ul pipette tip, a straight scratch was produced on the cell layer. Every 24 h, the quantification of wound closure was assessed. **E**, **F** Transwell assays showing the migration and invasion capacity of control and shUSP8 iCCA cells. **G**, **H** The ability of control and shUSP8 iCCA cells to proliferate is assessed by EdU staining assays. **I** The Xenograft Tumor Assay demonstrated that USP8 knockdown inhibited tumor development. The scatter diagram showed the tumor weight of the control and USP8-depletion group in 28 days after cell injection. The tumor volume was assessed every 2 days after the tumors were implanted. *P value < 0.05, **P value < 0.01, ***P value < 0.001

To assess the biological functions of USP8 in iCCA, we knocked down USP8 in HCCC9810 and RBE cells and overexpress USP8 in HCCC9810 cells. After USP8 depletion, the growth and clone formation capacity of iCCA cells was inhibitive in vitro (Fig. 5A, B). In contrast, USP8-overexpressing enhanced cell growth and colony formation ability (Figure S1A, B). Moreover, USP8 depletion restrained the cell migration ability in the scratch assay (Fig. 5C, D) while the results were opposite in USP8-overexpressing cells (Figure S1D). It indicated that migration and invasion ability of shUSP8 iCCA cells were weakened (Fig. 5E, F) but USP8-overexpressing promoted the migration and invasion ability by transwell assay (Figure S1C). In addition, we observed consistent experimental phenomenon in EdU cell proliferation assay in USP8-depletion (Fig. 5G, H) and USP8-overexpressing iCCA cells (Figure S1E).

What’s more, we used a xenograft model assay to explore whether USP8 affects tumor growth in vivo. The results showed knockdown USP8 significantly decreased tumor growth and USP8 overexpression enhanced tumor growth (Fig. 5I). These results convincingly revealed USP8 had a vital function in cell growth, colony formation, and tumor growth.

**Fig. 6:**
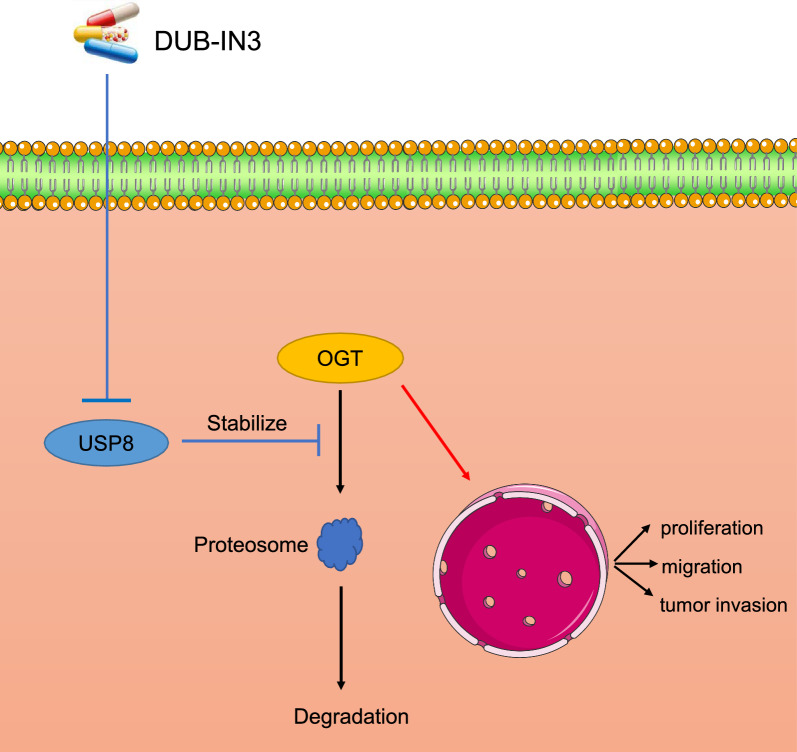
The mechanism of OGT regulation by USP8. USP8 stabilized OGT protein through deubiquitination manner

To further determine whether the functions of USP8 in regulating iCCA cells malignancy through the effects of OGT, we performed rescue experiments by overexpressing OGT in USP8 knockdown cells. We found that increased the expression of OGT facilitated the proliferation and clonogenicity of iCCA cells (Figure S2A, B). Meanwhile, it was indicated that re-expression of OGT mostly rescued the migration and invasion capacity of iCCA cells through the wound healing and transwell invasion assays (Figure S2C, E). Additionally, Edu assays indicated that the re-expression of OGT largely rescued the proliferation of HCCC9810 cells (Figure S2D). Taken together, above results suggested that USP8 promotes iCCA progression through OGT (Fig 6).

### Pharmacological inhibition of USP8 suppressed the malignancies and promoted iCCA cells’ response to pemigatinib

**Fig. 7:**
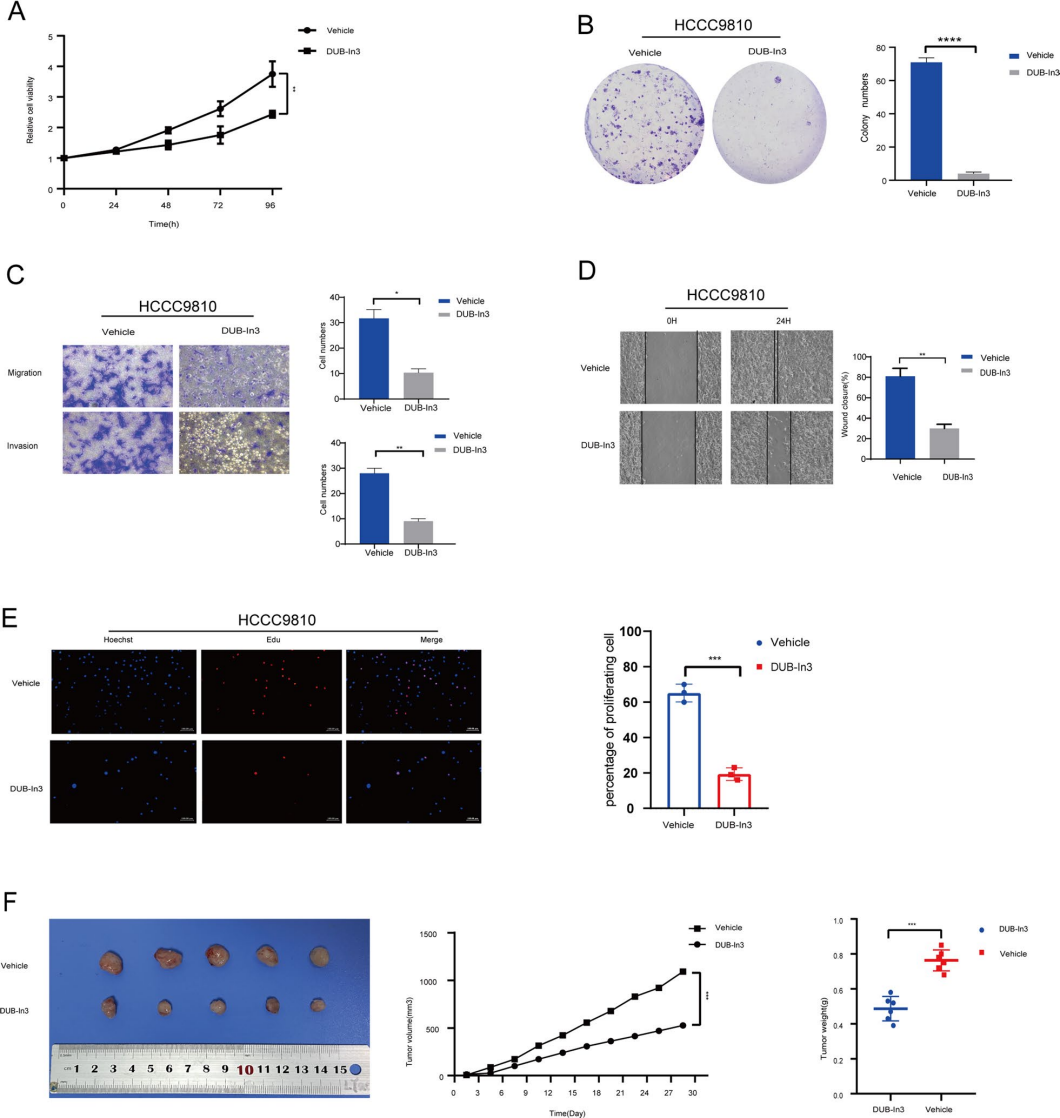
Inhibition of USP8 weakened the iCCA cells’ growth, colony formation, and tumor formation. **A** The vitality of cells was evaluated using the CCK8 assay in iCCA cells treated with DMSO or DUB-IN3 (5 μM). **B** Inhibition of USP8 decreases clone formation ability in HCCC9810 cells exposed to DMSO or DUB-IN3 (5 μM). **C**, **D** Transwell assays and wound healing assays showed the migration and invasion abilities of HCCC9810 cells treated with DMSO or DUB-IN3 (5 μM). E The vitality of cells was evaluated using EdU staining assays in iCCA cells treated with DMSO or DUB-IN3 (5 μM). F The Xenograft Tumor Assay demonstrated that treatment of DUB-IN3 inhibited tumor development. The scatter diagram showed the tumor weight of the vehicle and DUB-IN3 group. The tumor volume was assessed every 2 days after the tumors were implanted

In order to validate our findings, we used DUB-in3, a small molecule inhibitor of USP8, to perform some vitro experiments. We add either DUB-in3 (5 μM) or DMSO to the HCCC9810 cells. The cell proliferation capability was suppressed when treated with DUB-in3 (Fig. 7A). Correspondingly, colony formation ability, cell invasion, and cell migration were restrained under the treatment of DUB-in3 (Fig. 7B, C, and D). In the Edu assay, we noticed that DUB-in3 could also inhibit cell proliferation (Fig. 7E). In addition, we also validated whether USP8 inhibited tumor formation in vivo. We injected HCCC9810 cells into nude mice. After two weeks, the nude mice were randomly split into two groups. Then, DUB-in3 (5 μM) or DMSO was given with a dosage of 2 mg/kg of mouse body weight by intraperitoneal injection. We observed that DUB-in3 significantly suppressed tumor growth (Fig. 7F). Taken together, these results showed that inhibition of USP8 suppressed the tumor malignancies.

Besides, we validated whether inhibiting USP8 could improve the sensitivity of HCCC9810 cells to pemigatinib. We use DUB-in3, pemigatinib and their combination to treat HCCC9810 cells. It was indicated that DUB-in3 and pemigatinib could suppress the cell proliferation, respectively. And their combination showed stronger inhibition of cell proliferation (Figure S3 A and B). Meanwhile, consistent results were observed in colony formation and transwell assays (Figure S3 C, D and E). These findings indicated that inhibition USP8 promoted iCCA cells’ response to pemigatinib.

## Discussion

iCCA was a rare but highly malignant hepatobiliary tumor [29, 30]. In this study. We identify the USP8 is a β-catenin DUB that stabilizes OGT and promotes tumor growth, invasion, though its deubiquitylation activity. There is growing evidence that ubiquitination was crucial in the development of liver cancer [31]. Nevertheless, studies investigating the DUB in charge of OGT are few. In the present work, we found USP8 could remove ubiquitin from a substrate while also halt the degradation of the substrate. Moreover, USP8 was crucial for boosting cell growth and stimulating cells to enter the S-phase of the cell cycle [20]. USP8 also controlled protein trafficking and endocytosis, primarily by regulating the endosomal sorting complexes necessary for trafficking through its deubiquitination activity [32, 33]. Some evidence indicated USP8 was commonly overexpressed in human malignancies and was associated with poor overall survival in cancer patients [34]. USP8 protein was rarely expressed in normal cervical tissues, but its expression was increased in cervical squamous cell carcinoma (CSCC) tissues, especially in advanced CSCC [35]. Recently, Another study indicated that proliferation, viability, and colony formation of cholangiocarcinoma cells were all considerably decreased after USP8 silencing but it didn’t explain the deep mechanism of this phenomenon [25]. However, we first described the USP8 as a novel modulator that influenced the OGT ubiquitination, thus further influencing the malignancies of iCCA. First, we identified USP8 and OGT interacted with each other. Co-IP analysis identified the interaction between USP8 and OGT. Besides, USP8 decreased β-catenin polyubiquitination and promotes OGT protein stabilization. The expression level of OGT can be decreased by knocking down USP8, and the reduced OGT protein abundance could be restored by ectopic expression of USP8-WT. In addition, under the treatment of the proteasome inhibitor MG132, USP8 deletion could not further affect OGT protein level. USP8 depletion shortened the half-life time of OGT protein. Therefore, targeting USP8 might be an effective therapeutic strategy for iCCA.

According to previous research, USP8 commonly cleaved the K6, K48, and K63 links in ubiquitin chains [22]. In our study, we first demonstrated that the ubiquitin chain on OGT could be directly removed by USP8 in a dose-dependent manner. To further find out which type of ubiquitin chain on OGT was removed by USP8, we performed ubiquitination assay using a series of ubiquitin mutants, including K6, K11, K27, K29, K33, K48, and K63. The results suggested that USP8 eliminated the K27 and K48-linked ubiquitin chains from OGT, thereby preventing proteasome-mediated degradation of OGT. Lys48-linked polyubiquitination mostly targeted proteins for proteasome-mediated degradation, while the physiological roles of K27-linked polyUb polyurea were still poorly understood [36, 37]. Recent research had revealed that K27-linked polyUb was crucial for autoimmunity5 and DNA damage repair [38]. Meanwhile, overexpression of OGT could reverse the effect of USP8 knockdown on the malignancy of iCCA. These results indicated that USP8 could promote tumor proliferation, and invasion of iCCA through ubiquitinating OGT.

Post-translational modification (PTM) played a crucial role in iCCA oncogenesis. O-GlcNAcylation, a crucial type of PTM, was regulated by OGT and reversed by O-GlcNAcase (OGA). Previous studies reported OGT was involved in tumor formation and metastasis via regulating autophagy or ferroptosis in several cancers [39–41]. Altering O-GlcNAcylation levels might provide a new sight for curing fatal and chronic illnesses [42]. In this study, we found that overexpression of OGT can reverse the effect of knocking down USP8 on iCCA. USP8 may influence malignancy of iCCA through OGT. Therefore, a deeper understanding of the OGT upstream regulation mechanism could assist to identify novel therapeutic targets for certain malignancies.

Most patients had already missed the best time for surgery when they were first diagnosed. Systemic therapy was crucial in iCCA [11, 43]. As the results indicated depletion of USP8 led to decreased expression of OGT. Then, we used the DUB-in3, the targeted inhibitor drug of USP8, together with Pemigatinib to feed the nude mouse with xenograft tumor. The results showed that the therapeutic effect of the combination drug was better than that of Pemigatinib alone. Pemigatinib was a vital part in targeted therapy of iCCA. However, the therapeutic effect was still far from satisfactory. At present, the latest clinical trial showed that pemigatinib for the treatment of iCCA achieved the best ORR of only 37% [43]. Therefore, improving the sensitivity of targeted drugs become an urgent problem to be solved. Therefore, using the DUB-in3 inhibitor might have great potential to improve the therapeutic efficacy of targeted therapy.

In summary, we identified USP8 as a DUB for OGT, which promotes iCCA malignancies through stabilizing OGT by its deubiquitination activity. Besides, inhibiting USP8 might suggest a new strategy for targeted therapy of iCCA.

### Supplementary Information

Supplementary Material 1. Figure S1 Overexpression of USP8 promoted cell growth, colony and tumor formation. A USP8 overexpression promoted the proliferation in RBE cells. B USP8 overexpression promoted clone formation capability in RBE cells. C, D Transwell assays and wound-healing assays of RBE cells. E Representative images of EdU assay of RBE cells. *P value < 0.05, **P value < 0.01, ***P value < 0.001.

Supplementary Material 2. Figure S2 Increased OGT expression reversed the effect of USP8 depletion. A Cell proliferation assay of HCCC9810 cells. B Clone formation assay of HCCC9810 cells. C Transwell invasion assay of HCCC9810 cells. D Representative images of EdU assay of HCCC9810 cells. E Wound-healing assay of HCCC9810 cells. *P value < 0.05; **P value < 0.01; ***P value < 0.001.

Supplementary Material 3. Figure S3 Inhibition USP8 promoted iCCA cells’ response to pemigatinib. A The CCK8 assay was used to assess the cell viability of HCCC9810 cells under the treatment with DUB-in3 (5 μM), pemigatinib (10.0 nM) or their combination. B Representative images of EdU assay of HCCC9810 cells under the treatment with DUB-in3, pemigatinib or their combination. C Colony formation assays were performed to detect the colony formation of HCCC9810 cells under the treatment with DUB-in3, pemigatinib or their combination. D, E Transwell assays showed the migration and invasion abilities of HCCC9810 cells under the treatment with DUB-in3, pemigatinib or their combination. *P value < 0.05, **P value < 0.01, ***P value < 0.001.

Supplementary Material 4.

Supplementary Material 5.

Supplementary Material 6.

Supplementary Material 7.

## Data Availability

All data generated or analyzed during this study are available from the corresponding author on reasonable request.

## References

[1] Brindley PJ, Bachini M, Ilyas SI, Khan SA, Loukas A, Sirica AE, Teh BT, Wongkham S, Gores GJ (2021). Cholangiocarcinoma. Nat Rev Dis Primers.

[2] Bertuccio P, Malvezzi M, Carioli G, Hashim D, Boffetta P, El-Serag HB, La Vecchia C, Negri E (2019). Global trends in mortality from intrahepatic and extrahepatic cholangiocarcinoma. J Hepatol.

[3] Bertuccio P, Bosetti C, Levi F, Decarli A, Negri E, La Vecchia C (2013). A comparison of trends in mortality from primary liver cancer and intrahepatic cholangiocarcinoma in Europe. Ann Oncol.

[4] Utada M, Ohno Y, Tamaki T, Sobue T, Endo G (2014). Long-term trends in incidence and mortality of intrahepatic and extrahepatic bile duct cancer in Japan. J Epidemiol.

[5] Mazzaferro V, Gorgen A, Roayaie S, Droz Dit Busset M, Sapisochin G (2020). Liver resection and transplantation for intrahepatic cholangiocarcinoma. J Hepatol.

[6] Razumilava N, Gores GJ (2014). Cholangiocarcinoma. Lancet.

[7] Zhu Y, Kwong LN (2020). Insights into the origin of intrahepatic cholangiocarcinoma from mouse models. Hepatology.

[8] Abou-Alfa GK, Sahai V, Hollebecque A, Vaccaro G, Melisi D, Al-Rajabi R, Paulson AS, Borad MJ, Gallinson D, Murphy AG (2020). Pemigatinib for previously treated, locally advanced or metastatic cholangiocarcinoma: a multicentre, open-label, phase 2 study. Lancet Oncol.

[9] Subbiah V, Iannotti NO, Gutierrez M, Smith DC, Féliz L, Lihou CF, Tian C, Silverman IM, Ji T, Saleh M (2022). FIGHT-101, a first-in-human study of potent and selective FGFR 1–3 inhibitor pemigatinib in pan-cancer patients with FGF/FGFR alterations and advanced malignancies. Ann Oncol.

[10] Hoy SM (2020). Pemigatinib: first approval. Drugs.

[11] Silverman IM, Hollebecque A, Friboulet L, Owens S, Newton RC, Zhen H, Féliz L, Zecchetto C, Melisi D, Burn TC (2021). Clinicogenomic analysis of -rearranged cholangiocarcinoma identifies correlates of response and mechanisms of resistance to pemigatinib. Cancer Discov.

[12] Wilkinson KD (2000). Ubiquitination and deubiquitination: targeting of proteins for degradation by the proteasome. Semin Cell Dev Biol.

[13] Bello AI, Goswami R, Brown SL, Costanzo K, Shores T, Allan S, Odah R, Mohan RD (2022). Deubiquitinases in neurodegeneration. Cells.

[14] Sun T, Liu Z, Yang Q (2020). The role of ubiquitination and deubiquitination in cancer metabolism. Mol Cancer.

[15] Shi D, Wu X, Jian Y, Wang J, Huang C, Mo S, Li Y, Li F, Zhang C, Zhang D (2022). USP14 promotes tryptophan metabolism and immune suppression by stabilizing IDO1 in colorectal cancer. Nat Commun.

[16] Kanner SA, Shuja Z, Choudhury P, Jain A, Colecraft HM (2020). Targeted deubiquitination rescues distinct trafficking-deficient ion channelopathies. Nat Methods.

[17] Lange SM, Armstrong LA, Kulathu Y (2022). Deubiquitinases: from mechanisms to their inhibition by small molecules. Mol Cell.

[18] Neutzner M, Neutzner A (2012). Enzymes of ubiquitination and deubiquitination. Essays Biochem.

[19] Katz EJ, Isasa M, Crosas B (2010). A new map to understand deubiquitination. Biochem Soc Trans.

[20] Mathieu J, Michel-Hissier P, Boucherit V, Huynh J-R (2022). The deubiquitinase USP8 targets ESCRT-III to promote incomplete cell division. Science (New York, NY).

[21] Zhao Y, Peng D, Liu Y, Zhang Q, Liu B, Deng Y, Ding W, Zhou Z, Liu Q (2022). Usp8 promotes tumor cell migration through activating the JNK pathway. Cell Death Dis.

[22] Xiong W, Gao X, Zhang T, Jiang B, Hu M-M, Bu X, Gao Y, Zhang L-Z, Xiao B-L, He C (2022). USP8 inhibition reshapes an inflamed tumor microenvironment that potentiates the immunotherapy. Nat Commun.

[23] Mossakowska BJ, Rusetska N, Konopinski R, Kober P, Maksymowicz M, Pekul M, Zieliński G, Styk A, Kunicki J, Bujko M (2022). The expression of cell cycle-related genes in -mutated corticotroph neuroendocrine pituitary tumors and their possible role in cell cycle-targeting treatment. Cancers (Basel).

[24] Peng H, Yang F, Hu Q, Sun J, Peng C, Zhao Y, Huang C (2020). The ubiquitin-specific protease USP8 directly deubiquitinates SQSTM1/p62 to suppress its autophagic activity. Autophagy.

[25] Jing X, Chen Y, Chen Y, Shi G, Lv S, Cheng N, Feng C, Xin Z, Zhang L, Wu J (2020). Down-regulation of USP8 Inhibits cholangiocarcinoma cell proliferation and invasion. Cancer Manag Res.

[26] Tang J, Long G, Hu K, Xiao D, Liu S, Xiao L, Zhou L, Tao Y: Targeting USP8 Inhibits O-GlcNAcylation of SLC7A11 to Promote Ferroptosis of Hepatocellular Carcinoma via Stabilization of OGT. Adv Sci (Weinh) 2023, 10(33):e2302953. 10.1002/advs.202302953 PMC10667802 37867237

[27] Chang Y-H, Weng C-L, Lin K-I (2020). O-GlcNAcylation and its role in the immune system. J Biomed Sci.

[28] Islam MT, Chen F, Chen H (2021). The oncogenic role of ubiquitin specific peptidase (USP8) and its signaling pathways targeting for cancer therapeutics. Arch Biochem Biophys.

[29] Jeon Y, Kwon SM, Rhee H, Yoo JE, Chung T, Woo HG, Park YN (2023). Molecular and radiopathologic spectrum between HCC and intrahepatic cholangiocarcinoma. Hepatology.

[30] Dong L, Lu D, Chen R, Lin Y, Zhu H, Zhang Z, Cai S, Cui P, Song G, Rao D (2022). Proteogenomic characterization identifies clinically relevant subgroups of intrahepatic cholangiocarcinoma. Cancer Cell.

[31] Liu J, Wu Z, Han D, Wei C, Liang Y, Jiang T, Chen L, Sha M, Cao Y, Huang F (2020). Mesencephalic astrocyte-derived neurotrophic factor inhibits liver cancer through small ubiquitin-related modifier (SUMO)ylation-related suppression of NF-κB/snail signaling pathway and epithelial-mesenchymal transition. Hepatology.

[32] Wright MH, Berlin I, Nash PD (2011). Regulation of endocytic sorting by ESCRT-DUB-mediated deubiquitination. Cell Biochem Biophys.

[33] Berlin I, Higginbotham KM, Dise RS, Sierra MI, Nash PD (2010). The deubiquitinating enzyme USP8 promotes trafficking and degradation of the chemokine receptor 4 at the sorting endosome. J Biol Chem.

[34] Byun S, Lee S-Y, Lee J, Jeong C-H, Farrand L, Lim S, Reddy K, Kim JY, Lee M-H, Lee HJ (2013). USP8 is a novel target for overcoming gefitinib resistance in lung cancer. Clin Cancer Res.

[35] Yan M, Zhao C, Wei N, Wu X, Cui J, Xing Y (2018). High Expression of ubiquitin-specific protease 8 (USP8) is associated with poor prognosis in patients with cervical squamous cell carcinoma. Med Sci Monit.

[36] Tracz M, Bialek W (2021). Beyond K48 and K63: non-canonical protein ubiquitination. Cell Mol Biol Lett.

[37] Sun Z, Lu H, Xiao W, Li Y, Xu P (2020). Progress in K27 ubiquitin modification. Sheng Wu Gong Cheng Xue Bao.

[38] Zhou Q, Zhang J (2022). K27-linked noncanonic ubiquitination in immune regulation. J Leukoc Biol.

[39] Lu Q, Zhang X, Liang T, Bai X (2022). O-GlcNAcylation: an important post-translational modification and a potential therapeutic target for cancer therapy. Mol Med.

[40] Liu Y-Y, Liu H-Y, Yu T-J, Lu Q, Zhang F-L, Liu G-Y, Shao Z-M, Li D-Q (2022). O-GlcNAcylation of MORC2 at threonine 556 by OGT couples TGF-β signaling to breast cancer progression. Cell Death Differ.

[41] Li X, Wu Z, He J, Jin Y, Chu C, Cao Y, Gu F, Wang H, Hou C, Liu X (2021). OGT regulated O-GlcNAcylation promotes papillary thyroid cancer malignancy via activating YAP. Oncogene.

[42] Yang X, Qian K (2017). Protein O-GlcNAcylation: emerging mechanisms and functions. Nat Rev Mol Cell Biol.

[43] Kelley RK, Bridgewater J, Gores GJ, Zhu AX (2020). Systemic therapies for intrahepatic cholangiocarcinoma. J Hepatol.

