# Supplementary figures and images for: USP8 promotes the tumorigenesis of intrahepatic cholangiocarcinoma via stabilizing OGT
Source: Cancer Cell Int. 2024 Jul 7;24:238. doi: 10.1186/s12935-024-03370-w (PMC11229306; doi:10.1186/s12935-024-03370-w)

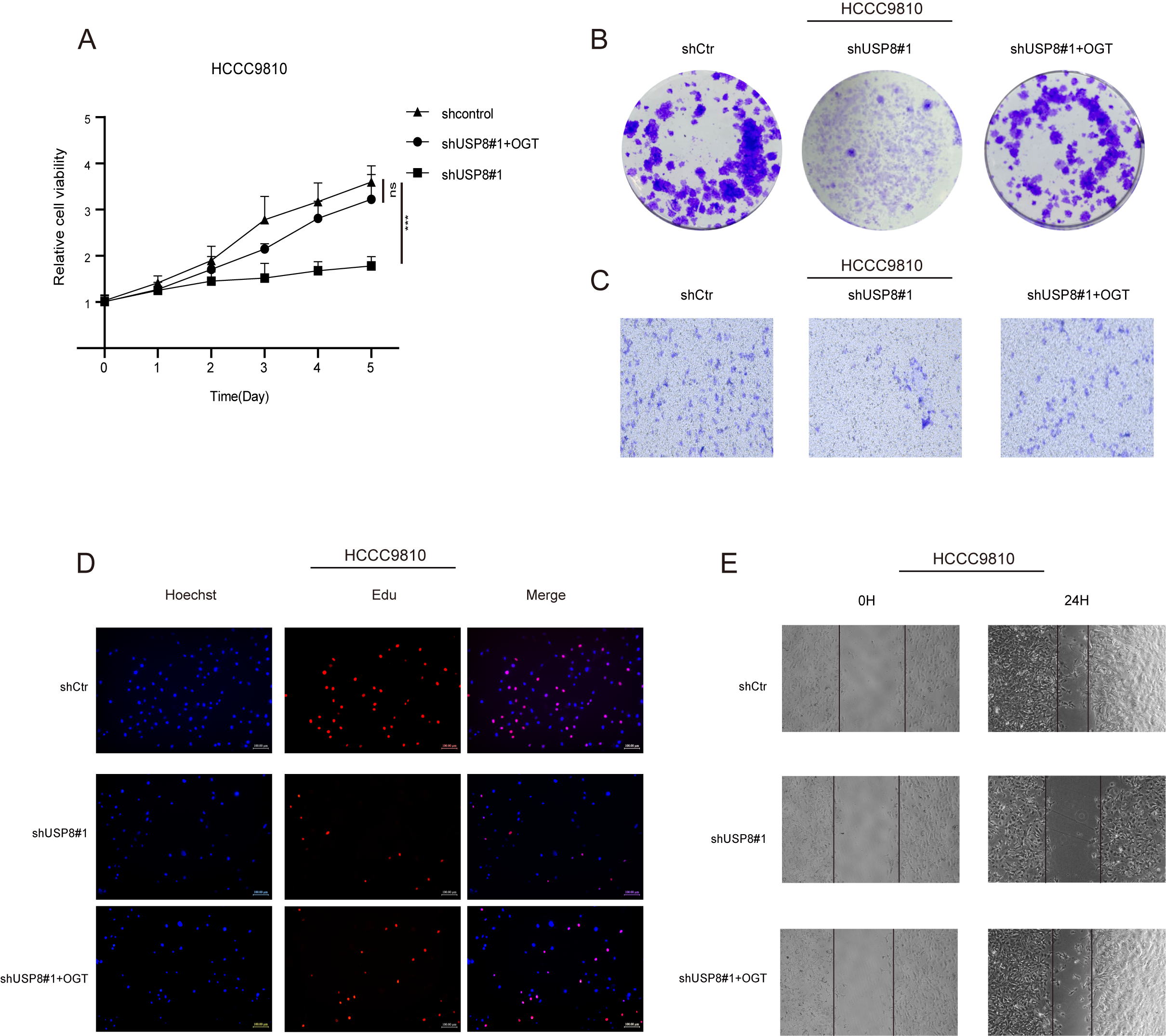

Supplement: Supplementary file 1 — Supplementary Material 1. Figure S1 Overexpression of USP8 promoted cell growth, colony and tumor formation. A USP8 overexpression promoted the proliferation in RBE cells. B USP8 overexpression promoted clone formation capability in RBE cells. C, D Transwell assays and wound-healing assays of RBE cells. E Representative images of EdU assay of RBE cells. *P value < 0.05, **P value < 0.01, ***P value < 0.001. [file 12935_2024_3370_MOESM1_ESM.tif]

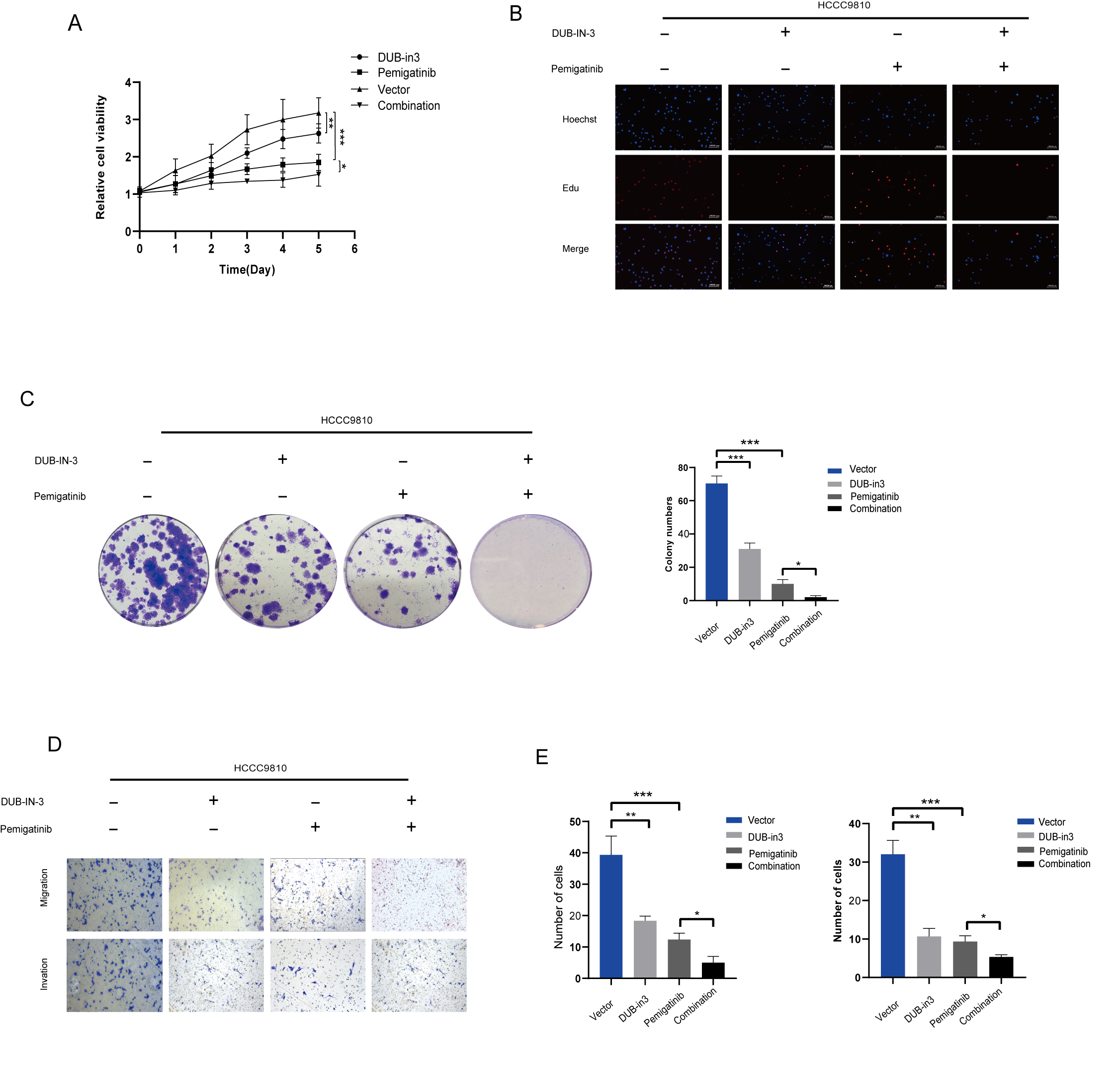

Supplement: Supplementary file 3 — Supplementary Material 3. Figure S3 Inhibition USP8 promoted iCCA cells’ response to pemigatinib. A The CCK8 assay was used to assess the cell viability of HCCC9810 cells under the treatment with DUB-in3 (5 μM), pemigatinib (10.0 nM) or their combination. B Representative images of EdU assay of HCCC9810 cells under the treatment with DUB-in3, pemigatinib or their combination. C Colony formation assays were performed to detect the colony formation of HCCC9810 cells under the treatment with DUB-in3, pemigatinib or their combination. D, E Transwell assays showed the migration and invasion abilities of HCCC9810 cells under the treatment with DUB-in3, pemigatinib or their combination. *P value < 0.05, **P value < 0.01, ***P value < 0.001. [file 12935_2024_3370_MOESM3_ESM.tif]
