# Supplementary material for: USP8 promotes the tumorigenesis of intrahepatic cholangiocarcinoma via stabilizing OGT
Source: Cancer Cell Int. 2024 Jul 7;24:238. doi: 10.1186/s12935-024-03370-w (PMC11229306; doi:10.1186/s12935-024-03370-w)
Supplement: Supplementary file 4 — Supplementary Material 4. [file 12935_2024_3370_MOESM4_ESM.docx]

| Variables | USP8 expression | | *P* value |
| --- | --- | --- | --- |
|  | High group (n = 67) | Low group (n = 59) |  |
| Age(years) | 59.626±(8.662) | 57.611±(9.920) | 0.255 |
| CA19-9(U/ml) | 208.731±(269.542) | 181.186±(265.181) | 0.565 |
| Tumor size(cm) | 6.856±(3.141) | 5.374±(2.922) | **0.007** |
| Gender |  |  | 0.608 |
| Male | 36 | 29 |  |
| Female | 31 | 30 |  |
| Hepatitis infection |  |  | 0.691 |
| Presence | 12 | 9 |  |
| Absence | 55 | 50 |  |
| Differentiation |  |  | 0.531 |
| I+II | 18 | 13 |  |
| III+IV | 49 | 46 |  |
| T stage |  |  | 0.136 |
| T1 | 35 | 36 |  |
| T2+T3 | 32 | 23 |  |
| Lymph node metastasis |  |  | 0.111 |
| Presence | 26 | 15 |  |
| Absence | 41 | 44 |  |
| TNM stage |  |  | **0.027** |
| I+II | 19 | 28 |  |
| III+IV | 48 | 31 |  |
