# Supplementary material for: USP8 promotes the tumorigenesis of intrahepatic cholangiocarcinoma via stabilizing OGT
Source: Cancer Cell Int. 2024 Jul 7;24:238. doi: 10.1186/s12935-024-03370-w (PMC11229306; doi:10.1186/s12935-024-03370-w)
Supplement: Supplementary file 7 — Supplementary Material 7. [file 12935_2024_3370_MOESM7_ESM.docx]

| Antibodies |  |  |  |
| --- | --- | --- | --- |
| USP8 Antibody | Cell Signaling Technology | #8728 |  |
| OGT Polyclonal antibody | Proteintech | Cat No. 11576-2-AP |  |
| MYC tag Monoclonal antibody | Proteintech | Cat No. 60003-2-Ig |  |
| DYKDDDDK tag Monoclonal antibody | Proteintech | Cat No. 66008-4-Ig |  |
| GAPDH Monoclonal antibody | Proteintech | Cat No. 60004-1-Ig |  |
|  |  |  |  |
| REAGENT |  |  |  |
| MG-132 | MedChem Express | Cat. No.: HY-13259 |  |
| Cycloheximide | MedChem Express | Cat. No.: HY-12320 |  |
| DUB-in3 | MedChem Express | Cat. No.: HY-50737 |  |
| CCK8 kit | MedChem Express | HY-K0301-100T |  |
| Cell-Light EdU Apollo567 In Vitro Kit(100T ) | Ruibobio | C10310-1 |  |
| RPMI 1640 | Cellmax | CGM112.05 |  |
| RPMI DMEM | Cellmax | CGM105.05 |  |
| FBS | Cellmax | SA201.02 |  |
| RIPA lysis buffer | Abiowell | AWB0136 |  |
| IP lysis buffer | Abowell | AWB0164 |  |
